# Absorption of Iron Naturally Present in Soy

**DOI:** 10.1016/j.advnut.2025.100396

**Published:** 2025-02-26

**Authors:** Laura S Hackl, Diego Moretti, Magalie Sabatier

**Affiliations:** 1 Nestlé Institute of Health Sciences, Nestlé Research, Société des Produits Nestlé S.A., Lausanne, Switzerland; 2 Nutrition Research, Swiss Distance University of Applied Sciences (FFHS)/University of Applied Sciences of South Switzerland (SUPSI), Zürich, Switzerland

**Keywords:** iron absorption, iron bioavailability, isotope studies, phytoferritin, soy concentrate, soy flour, soy isolate, soy protein, texturized soy

## Abstract

Plant-based foods can offer sustainable and healthy dietary choices. Soybeans and derivatives (for example, flour, concentrate, or isolate) are the primary protein source for plant-based products, including meat analogs, and are naturally rich in iron. To investigate the nutritional contribution of iron naturally present in soy, this narrative review presents iron bioavailability data from isotope studies in humans aged >3 y. To allow interstudy comparison, we adjusted mean iron absorption for iron status (that is, serum ferritin of 15 μg/L), quantified native iron absorbed, and compared with daily human requirements for absorbed iron where possible. Adjusted iron absorption from soybeans served as part of meals varied widely (4.1%–22.2%), translating to contributions of 13%–70% and 10%–40% to the daily requirements for absorbed iron in adult males and females, respectively. Similar results were found for meals prepared with soy flour (full fat, defatted, and texturized) and soy protein concentrates, whereas iron bioavailability from soy protein isolates may be reduced. Within a meal, partial substitution (≤30%) of meat with soy concentrates and isolates did not meaningfully impair total iron absorption. In all conditions, low phytic acid levels and co-ingestion of ascorbic acid improved the absorption of iron naturally present in soy. Available evidence suggests that soy-based products can provide a meaningful contribution to daily requirements for absorbed iron, especially if phytic acid is below defined thresholds to optimize absorption and/or if products include iron absorption enhancers such as ascorbic acid. Further research is needed to understand the factors affecting iron bioavailability from these products, especially the soy cultivar, the roles of phytoferritin and the protein profiles of different soy protein ingredients, as well as the processes to produce them. Long-term assessments of the impact of soybean-based products on iron status are also warranted.

**Statement of significance**

This narrative review is the first evidence synthesis on iron bioavailability from soy and soy derivatives in predominantly adult, human populations. Findings indicate that native iron from soybeans, soy flours, and soy concentrates, and to a smaller extent isolates, can represent a relevant source of absorbable dietary iron.

## Introduction

Aligning with the United Nations Sustainable Development Goals and implementing the Paris Agreement requires a substantial global dietary transition toward sustainable diets. The EAT-Lancet Commission suggests that by 2050, a dietary pattern that promotes human and planetary health involves a doubling of the consumption of fruits, vegetables, legumes, and nuts, with a simultaneous reduction in the intake of added sugars and red meat especially in high-income countries [1,2]. In addition to environmental and health arguments, plant-based diets, including meat analogs or substitutes may also be preferred for animal welfare, ethical, economic, and spiritual reasons [3]. Health benefits linked to plant-based diets are associated with a reduced risk for hypertension and cardiovascular disease, improvements in the prevention and management of overweight and related conditions, and offer additional mental and physical health benefits [4].

Although nutrient inadequacies can exist across all dietary patterns, including vegan, vegetarian, and meat-based diets [5,6], iron is considered at risk of shortfall for consumers of plant-based diets. However, available evidence is often dated, and many studies have low sample sizes and do not consider geographic context [5,7]. Two types of dietary iron exist in omnivorous diets: nonheme and heme iron. Legumes are among the core pillars of plant-based diets and only contain nonheme iron. Nonheme iron absorption can be decreased by inhibitors for example, phytic acid, some polyphenols but also increased by enhancers such as ascorbic acid (AA, that is, vitamin C) [8]. Iron absorption from heme iron, present in meat, fish, and poultry is substantially higher than from nonheme iron and is not impacted by the presence of dietary absorption inhibitors and enhancers [8].

The Institute of Medicine estimates iron absorption from omnivorous diets at ∼18% [9], and more recent analyses indicate a lower proportion of bioavailable iron in the United States diet of 15.1%–15.5% [10]. Although the WHO suggests adjusting the recommended dietary allowance (RDA) for iron in relation to the dietary composition and the respective bioavailability [11], the United States Institute of Medicine proposed a 1.8 times higher RDA for iron intake in vegetarians compared with individuals consuming meat [9]. In contrast, the European Food Safety Authority does not differentiate its dietary recommendations for iron according to dietary patterns [12].

In line with the shift toward more sustainable (plant-based) diets, there is an increasing interest in legumes and legume-based ingredients to produce plant-based products and meat analogs. Currently, soybeans are the primary protein source for such products in part driven by their beneficial nutritional profile with high-quality protein and fat contents. In addition, soybean production requires only very limited agronomic nitrogen fertilization, which can be an important consideration in sustainable agriculture [13]. Soy protein ingredients include whole soybeans, full-fat or defatted soy flour, as well as soy protein concentrates and isolates. Soy flour is prepared from white flakes (dehulled and defatted soybeans) that are milled to varying degrees. Soy protein concentrates and isolates are prepared from white flakes or soy flour via extraction and drying procedures. Nutrient contents in the different products are process-dependent and subject to variability [14]. The highest protein levels can be found in soy protein isolate (92%) followed by concentrates (67.5%), and lowest in soy flour ranging between 42% and 52% [13]. Similarly, soy protein isolates have the highest proximate iron content (16 mg/100 g), compared with defatted soy flour (1 1mg/100 g) and soy protein concentrate (10 mg/100 g) [15]. The USDA indicates lower iron contents for defatted (7.5 mg/100 g) and full-fat (9.5 mg/100 g) soy flour [16].

This narrative review synthesizes available evidence on the human bioavailability of iron naturally present in soy. We considered evidence that was generated in human subjects, aged 3 y and above, in which iron absorption was assessed using isotope methodologies. Studies reporting on iron absorption from iron salts used for iron fortification of a soy-based food matrix, soy sauce, or fermented products, or studies not reporting on the levels of native iron present in soy were not included. To contextualize the findings, we performed additional searches to further understand the form of iron naturally present in soy. We classified studies (*N* = 22, Table 1) [[17], [18], [19], [20], [21], [22], [23], [24], [25], [26], [27], [28], [29], [30], [31], [32], [33], [34], [35], [36], [37]] by the type of soybean ingredient (soybean or derivative) and meal type (soybean or derivative as main meal component or as extender/replacer of meat) as administered.

**TABLE 1:** Overview of studies in which absorption of iron naturally present in soybean and its derivatives was assessed^1^.

Study	Population^1^ *N*	Meal/comparison	Native Fe/total Fe (mg) label method	Phytic acid content PA:Fe, molar ratio	Nonheme iron absorption Geo. Mean, % (range)^2^	Comment
Iron absorption from soybeans served as a meal.						
Layrisse et al. 1969 [22]	*N* = 17 5 iron deficient (ID) Country: nr	Bean mush: soybeans (Hawkeye) and conventional soybean flour boiled separately for 15 min, proportionally mixed (25–40 g labeled, 10–25 g conventional), subsequently baked (60 min) at 150°C.	4 mg native incl. tracer Intrinsic (I): 5 μCi ^59^Fe	nr	11.3 (1.5–42.2)	Geo mean Fe absorption from ferrous ascorbate reference: 23.8%, indicating no Fe deficiency [17]
Börn-Rasmussen et al. 1973 [19]	*N* = 15 (3F/12M) Country: nr	Bean mush: Labeled soybeans finely ground, mixed with conventional soybean flour; subsequently boiled (15 min) and baked (25 min) at 210°C.	2.5 mg native incl. tracer I: 3 μCi ^55^Fe Extrinsic, radioactive [E(R)]: 1 μCi ^59^Fe as ferric chloride (FeCl_3_)	nr	I: 2.6 (0.2–9.4) E: 2.7 (0.3–9.6)	–
Murray-Kolb et al. 2003 [18]	*N* = 18 all F ID, 11 received soup; 7 received the muffin (*N* = 7) Country: United States	Labeled whole soybeans (Tokyo) baked (1 h) at 149°C. Soup: 47 g whole soybean + chicken bouillon. Muffin: 23 g soybean flour + baking powder, sugar, cream of tartar, salt, shortening, milk; baked (15 min) at 177°C.	Soup: 4.5 mg native incl. tracer I: 2 μCi ^55^Fe Muffin: 3 mg native incl. tracer I: 1 μCi ^55^Fe	Soybean (raw): 31.77 μmol/g Soup: 18.3:1 Muffin: 13.7:1	24.5 (9–36) No significant difference between Fe absorption from soup or muffin.	Geo mean Fe absorption from ferrous ascorbate reference: 57.3% (range: 26%–84%), reflecting Fe deficiency [17]. Significant inverse correlation between serum ferritin (SF) and Fe absorption.
Cook et al. 1972 [20]	*N* = 11 (6 ID) Country: Venezuela and/or United States	Bean mush: Prepared as in [22], whereas labeled food was mixed with 2-4 times its weight of unlabeled food.	3 mg native incl. I: 10 μCi ^58^Fe Extrinsic, stable [E(S)]: 0.1 mg as FeCl_3_	nr	I: 6.3 (0.2–21.1) E: 7.8 (0.3–30.1)	Geo mean Fe absorption from ferrous ascorbate reference: 42.2%, reflecting Fe deficiency [17].
Sayers et al. 1973 [21]	*N* = 10 (mainly F, all ID) Country: nr	Biscuit: 20 g (labeled + conventional) whole soybeans (Hawkeye) boiled and baked (45 min) at 205°C. Biscuit + AA (subset *n* = 5): addition of 100 mg AA prior cooking, AA:Fe after baking 2.6:1.	2.6 mg native incl. tracer E(R): 1.5 μCi ^59^Fe +2.0 mg Fe as FAC	nr	Biscuit I: 15.5 (7.1–48.0) E: 16.7 (49.9–7.9) Biscuit + AA I: 9.8 (1.0–23.9) E: 26.7 (1.3–26.7)	The study found no effect of AA on Fe absorption; data were not normalized for subjects’ Fe status.
Lynch et al. 1984 [24]	*N* = 10 (all M) Country: USA	Soup: 100 g cooked soybeans + 50 g broth (bay leaves, garlic powder, onion, margarine, salt, red pepper).	2.7 mg native E(S): 1.3 mg as FeCl_3_	nr	1.7 (0.3–9.81)	Geo mean Fe absorption from ferrous ascorbate reference: 16.3%, reflecting no Fe deficiency [17].
Morck et al. 1982 [23]	Study 1 *N* = 9 (all M) Country: United States	Whole soybean (WSB) meals (boiled vs. baked) vs. isolated soy protein (ISP) slurry (uncooked vs. baked). WSB boiled (100 g): WBS soaked overnight, then boiled (2 h) and served after decantation with diced onions, salt, bay leaves monosodium glutamate. WSB baked (38 g): 100 g WBS baked at 200°C, 45 min. ISP was served as a semisynthetic drink made of corn sirup and oil with 16.9 g ISP (14.7 g protein). ISP was added either uncooked or after baking (1 h at 200°C).	WSB boiled: 1.3 mg native + E(R): 1.7 mg as FeCl_3_ WSB baked: 0.5 mg native Fe + E(R): 2.5 mg as FeCl_3_ ISP (both): 2 mg native + E(R): 2 mg as FeCl_3_ FeCl_3_ was labeled with either 2 μCi ^59^Fe or 5 μCi ^56^Fe.	nr	WSB boiled: 1.06 ±1 SE: 0.80; 1.41 WSB baked: 1.60 ±1 SE: 1.26; 2.02 Absorption ratio baked vs. boiled WSB: 1.50 (*P* < 0.05). Uncooked ISP: 0.64 ± 1 SE: 0.53; 0.78 Baked slurry: 1.28 ± 1 SE: 1.08; 1.52 Absorption ratio baked slurry/slurry: 1.99 (*P* < 0.001).	Subjects were not Fe deficient (mean serum ferritin: 46 μg/L).
Nonheme iron absorption from meals containing soy vs. meat as protein source.						
Etcheverry et al. 2006 [25]	*N* = 26 4–8 y (18F/8M) Country: United States	Soy chili: 52 g soy crumbles (soy protein concentrate) (*N* = 14) Beef chili: 40 g ground roast beef (*N* = 12) Either meal served with corn chips, grated mild cheddar cheese and seasoned tomato.	Soy chili: 2.3 mg native + E(S): 1 mg ^58^FeSO_4_ Beef chili: 0.9 mg native nonheme Fe + E(S): 1.4 mg as FeSO_4, +_ 1 mg ^58^FeSO_4_	Soy chili: 3.7:1 Beef chili: 3.5:1	Soy chili: 3.5 Beef chili: 7.6 (*P* = 0.0015)	Nonheme native Fe quantities absorbed (not accounted for heme Fe absorption): Soy chili: 0.082 mg Beef chili: 0.068 mg∗
Hallberg et al. 1984 [27]	*N* = 39 (15F/24M) not anemic Country: Sweden	Basal meal (maize chapatti, black beans, rice) served with either: - 75 g meat (*N* = 9) - 33 g defatted soy flour∗ (*N* = 10) - 125 g cauliflower as source of ascorbic acid (AA; 65 mg); AA:Fe 3.8:1 (*N* = 10) - 50 mg pure AA; AA:Fe 3.7:1 (*N* = 10). Soy flour was mixed into the chapatti dough.	E (R): 0.6 mg Basal: 4.7 mg native; Meat: 5.3 mg nonheme, 0.7 mg heme Soy flour: 10.7 mg native Cauliflower: 5.4 mg native AA: 4.3 mg native Meals were labeled with 1.5 μCi ^59^Fe or 2 μCi ^55^Fe	Basal: 4:1 + Meat: 4:1 + Soy flour: 3.2:1 + cauliflower: 4:1 + AA: 4:1	Absorption (%); absorbed Fe (mg) Basal 3.5; 0.17 + Meat∗: 8.0; 0.45 + Soy flour: 4.0; 0.51 + Cauliflower: 7.7; 0.58 + AA: 3.4; 0.41 Fe absorption in basal vs. other meals *P <* 0.01.∗Does not account for heme Fe absorption.	Increase in Fe absorption in soy flour meal attributed to Fe present in soy flour. Absorption (%) adjusted to 40 % reference dose absorption ±SEM: Basal: 3.2 ±1.2 + Meat: 8.4 ±2.0 + Soy flour: 4.8 ±1.2 + Cauliflower: 10.8 ±3.6 + AA: 2.8 ±0.64
Nonheme iron absorption from native iron in soybean derivatives obtained by food processing						
Morck et al. 1982 [23]	Study 2 *N* = 7 All M Country: United States	ISP was served as a semisynthetic liquid meal (SLM) made of corn sirup and oil with 16.9 g ISP or 18.4 g of egg albumin (EA) (corresponding to 14.7 g protein). Both types of SLM were served with and without AA. - SLM-ISP + AA: 100 mg AA - SLM-EA + AA: 100mg AA AA:Fe : 7.6:1	SLM-ISP: 2 mg native + E: 2 mg as FeCl_3_ SLM-EA: 0.1 mg native +E: 3.9 mg as FeCl_3_ labeled with either 2 μCi ^59^Fe or 5 μCi ^56^Fe.	nr	SLM-ISP: 0.56 ± 1 SE: 0.47; 0.66 SLM-ISP + AA: 3.20 ± 1 SE: 2.66; 3.84 SLM-EA: 5.05 ± 1 SE:4.31; 5.91 SLM-EA + AA: 10.19 ± 1 SE: 9.14; 11.37	Subjects were not ID (mean serum ferritin: 31 μg/L). Absorption ratio SLM-ISP +AA/ SLM-ISP: 5.69 (*P* < 0.001). Absorption ratio SLM-EA +AA/ SLM-EA: 2.2 (*P* < 0.001). Absorption ratio SLM-ISP+AA/ SLM-EA: 0.63 (n.s.)
Hurrell et al. 1992 [28]	*N* = 32 Country: United States	Studies 1–4: meals were SLM containing 67 g hydrolyzed corn starch, 36 g corn oil, 12 mL vanilla extract, 200 mL water, and 30 g protein differing in Studies 1 through 4 as follows:	Studies 1–4: E(R) Range of native Fe content in ISP: 0.130-0.180 mg/g			Soy flours were the same within each study, batches differed in studies 1 and 2; studies 3 and 4 used the same soy flour batch.
	Study 1 *N* = 8 (2F/6M)	- ISP with native PA (meal 1A) - Dephytinized ISP (meal 1B) - Egg white (control 1)	Fe adjusted to 5.7 mg E: 3kBq ^59^FeCl_3_ or ^55^FeCl_3_	ISP Meal 1A: 4.9:1 ISP Meal 1B: 0.1:1	- Meal 1A: 1.50 - Meal 1B: 3.15 - Control 1: 6.34	1A vs. control 1 (*P <* 0.05) 1A vs. 1B (*P* <0.05)
	Study 2 *N* = 9 (4F/5M)	- ISP with native PA (2A) - Dephytinized ISP (acid-salt washing and ultrafiltration; 2B) - Restored PA ISP (2C) - Egg white (control 2)	s. above	2A: 4.4:1 2B: 0.7:1 2C: 6.1:1	- 2A: 0.92 (0.65–1.32) - 2B: 1.91 (1.34–2.71) - 2C: 1.08 (0.75–1.54) - Control 2: 5.75 (3.96–8.33)	2A, 2B, 2C vs. Control 2 (*P* < 0.001) 2A vs. 2B (*P* < 0.05)
	Study 3 *N* = 8 (1F/7M)	- ISP with native PA (3A) - Dephytinized ISP with enzyme digestion (3B) - Restored PA ISP (3C) - Egg white (control 3)	s. above	3A: 5.0:1 3B: <0.1:1 3C: <0.1:1	- 3A: 0.53 (0.41–0.68) - 3B: 2.50 (2.10–2.97) - 3C: 0.78 (0.52–1.15) - Control 3: 5.48 (3.63–5.94)	3A, 3C vs. control 3 (*P* < 0.001) 3B vs. control 3 (*P* < 0.05) 3A vs. 3B (*P* < 0.01)
	Study 4 *N* = 7 (4F/3M)	- ISP with native PA (4A) - Dephytinized ISP (acid-salt washing and ultrafiltration: 4B) - Dephytinized ISP (enzyme treatment and ultrafiltration 4C) - Egg white (control 4)	s. above	4A: 3.5:1 4B: 0.2:1 4C: <0.1:1	- 4A: 1.36 (0.94–1.98) - 4B: 4.17 (3.01–5.76) - 4C: 5.48 (4.16–7.21) - Control 4: 9.72 (7.56–12.51)	4A vs. control (*P* < 0.001) 4B vs. control (*P* < 0.01) 4A vs. B (*P* < 0.001) 4A vs. C (*P* < 0.05)
Cook et al. 1981 [26]	*N* = 10 (all M) Country: United States	Study 2: SLM containing 68 g dextrimaltose, 35 g fat from corn oil + 29.4 g protein from either: - Full-fat soy flour (FSF), - Textured soy flour (TSF), - ISP, or - Egg albumin (control)	E (R) Native iron: • FSF: 3.4 mg, • TSF: 3.3 mg • ISP: 2.0 mg • Control: 0.1 mg Fe in all meals adjusted to 4 mg with FeCl_3_.	nr	- FSF: 0.97 (±1 SE: 0.77; 1.23) - TSF: 1.91 (±1 SE: 1.60; 2.27) - ISP: 0.41 (±1 SE: 0.31; 0.54) - Control: 5.5 (±1 SE: 4.42; 6.83)	TSF vs. FSF or ISP (*P* < 0.01) FSF vs. ISP (*P* < 0.01)
Reddy et al. 1996 [30]	Study 3 *N =* 9 (4F/5M) Nonanemic subjects, 3 subjects had SF <12 μg/L Country: United States	SLM (67 g hydrolyzed corn starch, 36 g corn oil, and 12 mL vanilla extract) mixed with - SLM + 300 mg PA as sodium phytate - SLM + 30 g phytate-free ISP - SLM + 300 mg PA as sodium phytate + 30 g phytate-free ISP - SLM only (Control)	E(R) Native: 5.2 mg Fe Native Fe from SLM was not reported including 37 kBq ^59^FeCl_3_ or 74 kBq ^55^FeCl_3_	Phytate content in ISP <0.01 mg/g	- SLM + PA: 1.82 (1.36–2.43) - SLM + ISP: 2.60 (2.01–3.26) - SLM + PA + ISP:1.22 (0.92–1.64) - Control: 11.44 (9.46–13.83)	SLM + PA vs. Control (*P <* 0.0001) SLM + ISP vs. Control (*P <* 0.001)
Lynch et al. 1994 [29]	*N =* 6 (3F/3M)	SLM (67 g hydrolyzed maize starch, 36 g corn oil, 12 mL vanilla extract, 200 mL water, and 30 g protein) either as - ISP - Hydrolyzed ISP: HP1 (amino: total nitrogen 0.19) - Hydrolyzed ISP: HP2 (amino: total nitrogen 0.47) - Egg white (control)	Native Fe: - ISP: 5.2 mg - HP 1: 6.7 mg - HP 2: 0.9 mg + E(R): 37 kBq ^59^FeCl_3_ or 111 kBq ^55^FeCl_3_ + Adjustment to a total of 7.2 mg Fe/serving	Phytic acid content in ISP: 1.70 % HP1: 0.23 % HP2: < 0.05	- ISP: 0.28 - HP 1: 1.86 - HP 2: 5.33 - Control: 3.10	ISP vs. HP 1 or 2 (*P* < 0.01)
Istfan et al. 1983 [31]	*N =* 6 (all M) Country: United States	Liquid formula provided over 82 d containing glucose, corn oil, salt, cellulose, potassium phosphate, vitamins and mineral premix, water, and soy concentrate (9% liquid weight). Incorporated in the diet as sole source of protein to supply 0.8 g /kg body weight. AA:Fe of 5.6:1	Fecal monitoring, E(S): ^58^FeCl_3_ was added to the liquid formula and consumed in 2 consecutive meals. 11.8 mg Fe only provided by soy concentrate	0.3% phytate phosphorus PA:Fe:1.8:1	9%–15% (Fe absorption was measured 7 times at 12-d intervals throughout the study.	Decline in SF; authors partly attributed this to blood withdrawals.
Istfan et al. 1983 B [37]	*N =* 8 (all M)	The formula composition was the same as in Istfan et al. [31] (0.65 g protein/kg body weight.) and contained either: soy concentrate or nonfat dry milk (control). The formula was the only source of protein incorporated into the meals.	Fecal monitoring, E(s) 2.5 mg Fe as ^58^FeCl_3_, divided equally in 6 consecutive meals administered over 2 d. Constant total Fe intake of ∼15 mg/d	nr	Mean ± SEM Soy formula: 28 ±7 Milk-based formula: 32 ±8	n.s.; authors reported that high Fe absorption is notable and clearly due to the high AA level of each meal (72 mg) supplied by the apple juice used as energy source
Nonheme iron absorption from meals in which soy was used as meat extender or replacer.						
Sandström et al. 1986 [33]	*N =* 8 ileostomized subjects (5 ID) Country: Sweden	Fe absorption was evaluated from diets for 2×24 h with meat as protein source or 25% meat replaced by soy flour, soy protein concentrate (SPC)] or ISP, resulting in 4 diet types - Meat diet (no soy protein) - Meat/soy diet: 17.5 g protein from soy flour or SPC or ISP.	Mass balance study	PA:Fe in - SF: 15.6:1 - SPC: 11.9:1 - ISP: 6.2:1	Apparent Fe absorption μmol ± SD from - Meat diet: 29.8 ±20.6 - Meat/SF: 24.2 ±22.9 - Meat/SPC diet: 38.2 ±17.9 - Meat/ISP diet: 46.1 ±38.5	The authors noted no obvious effect of replacing 25% of meat protein with soy in the diet.
Morris et al. 1987 [32]	*N =* 27 (all M) Country: United States	Fe absorption determined from a meal, that is, 150 g mashed potatoes, 50 g bread, 5 g butter, 120 g canned peaches, 244 g whole milk, patty (4 oz. uncooked). The patty contained either: - 100 % ground beef, or - 80% beef +20% soy protein, that is, either TSP, SPC, or ISP.	E(R); native Fe: - Beef: 1.9 - Beef + ISP or SPC: 2.3 - Beef + TSF: 2.6+ ^59^FeSO_4_ or^55^FeCl_3 +_ 3 mg as FeSO_4_	nr	- Beef: 1.31±SEM: 0.87; 1.97 - ISP: 0.90 ±SEM: 0.64; 1.27 - SPC: 1.30 ±SEM: 1.06; 1.58 - TSF: 0.88 ±SEM: 0.68; 1.13	Nonheme Fe absorption at 40% of reference dose Beef: 3.56 ±SEM: 5.04; 2.51 ISP: 5.09 ±SEM: 7.39; 3.51 SPC: 4.21 ±SEM: 5.65;3.40 TSF: 3.45 ±SEM: 3.95; 3.01 n.s. between conditions
Woodhead et al. 1988 [34]	*N =* 16 (8F/8M) Children aged 7–10 y Country: United States	Fe absorption determined from meal that is, 28 g hamburger bun, 14 g tomato ketchup, 28 g raw carrot sticks, 10 g chocolate chip cookies, 15 g potato chips, 240 g cocoa milk, and 50 g patty containing either: - 100% beef or - 70% beef + 30% soy (type nr).	E(S) 1 mg ^58^FeSO_4_ administered with cocoa milk. Native Fe: beef lunch: 3.44 Beef/soy lunch: 3.83	nr	- Beef: 2.0 ±1 SD: 1.0;4.1 - Beef + soy: 1.1 ±1 SD: 0.4;2.6 Beef vs. Beef + soy (*P <* 0.05).	Nonheme Fe absorption (mg/d) Beef (0.08) vs. beef + soy (0.05; n.s.). ≈0.03 mg/d: Authors’ conclusion: absolute difference may not be nutritionally relevant.
Cook et al. 1981 [26]	Study 3 *N =* 11 (all M) Country: United States	Meals containing 55 g French fried potatoes, 180 mL vanilla milkshake, bun and a meat patty containing either: - 100 g precooked lean ground beef - 100 g beef + 30 g dry TSF (protein ratio 1.2:1) - 70 g beef + 30 g dry TSF (protein ratio:0.8:1). Patties were broiled for 5–7 min per side.	E(R) 0.1 mg Fe as ^59 or 55^ FeCl_3_ administered with the milkshake.	nr	- Beef :3.2 ±1 SE: 2.43;4.21 - Beef + soy (3:1): 1.24 ±1 SE: 0.89;1.72 - Beef + soy (2:1): 1.51 ±1 SE: 1.06;2.17 Beef + soy meals differed from beef meal (*P <* 0.001).	Geo mean Fe absorption from ferrous ascorbate reference: 19.9%., indicating no Fe deficiency [17].
Hallberg et al. 1982 [35]	Study 3 *N =* 10 (1F/9M) not anemic Country: Sweden	In study 3 and 4: Hamburger meal containing 60 g string beans, 150 g mashed potatoes, 82 g patty, consisting of: - 100% minced meat or - 50% meat + 50% TSF.	E(R) Meat: 0.5 mg heme Fe, 3.0 mg nonheme Fe. Meat + soy: 0.25 mg heme, 3.8 mg nonheme Fe	Meat + soy: 1.6:1	- Meat: 12.9 - Meat + soy: 8.2 Meat vs. Meat + soy: n.s.	Geo mean Fe absorption of reference dose 40.9%. The total amount of nonheme Fe absorption from meat + soy > 100% meat condition (0.27 vs. with 0.20 mg), which the authors attribute to the high Fe content in TSF.
	Study 4 *N =* 9 (7F) not anemic Country: Sweden	Same as study 3, patty consisting of either: - 50% meat + 50% DSF, or - 50% meat + 50% dephytinized DSF.	Meat-DSF: 0.25 mg heme, 3.9 mg nonheme Fe Meat-dephytinized DSF: 0.25 mg heme, 3.5 mg nonheme Fe	Meat + DSF: 1.5:1 Meat + dephytinized DSF: 0	- Meat + soy: 4.9 - Meat + dephytinized soy: 4.4 Meat + soy vs. Meat + dephytinized soy: n.s.	Geo mean Fe absorption of reference dose 35.8%.
Lynch et al. 1985 [36]	Study 1 *N =* 9 (all M) Country: United States	Ground lean beef patty, broiled for 5–7 min, consisting of either: - 100 g beef, or - 70 g beef + 75 g hydrated TSF.	E(R) Beef patty: 0.9 mg nonheme Fe, 1.2 mg as heme Beef + soy patty: 5.3 mg nonheme Fe, 0.8 mg as heme 1 μCi ^59^FeCl_3_ for nonheme Fe absorption 3 μCi ^55^Fe hemoglobin for heme Fe absorption determination	nr	Nonheme Fe absorption: Beef patty: 24.82 ±1 SE: 21.09; 29.22 Beef + soy patty: 0.87 ±1 SE: 0.71; 1.01 Heme Fe absorption: - Beef patty: 17.03 ±1 SE: 14.81; 19.59 - Beef + soy patty: 27.13 ±1 SE: 23.79; 30.95	Fe absorption ratios differed significantly (*P <* 0.01). Marked reduction of nonheme Fe and increased of heme Fe absorption with soy.
	Study 2 *N =* 12 (all M) 2 subjects with depleted Fe stores Country: United States	Meal containing white bun, 68 g French fries, 145 mL milkshake, and patty broiled for 10 min and contained either: - 100 g beef or - 70 g beef + 30 g hydrated soy flour.	E(R) Beef meal: 1.8 mg, 1.2 mg as heme Beef + soy meal: 5 mg, 0.8 mg as heme 1 μCi ^59^FeCl_3_ for nonheme Fe absorption 3 μCi ^55^Fe hemoglobin for heme Fe absorption determination	nr	Nonheme Fe abs: - Beef patty meal: 5.05 ±1 SE: 3.54; 7.20 - Beef + soy patty meal: 1.90 ±1 SE: 3.54; 7.20 Heme Fe absorption: - Beef patty meal: 33.10 ±1 SE: 28.36; 28.63 - Beef + soy patty meal: 42.10 ±1 SE: 35.98; 49.26	Fe absorption ratios (Beef + soy/Beef meals) differed significantly (*P <*0.01).
	Study 3 *N =* 10 (all M) 1 subject with depleted Fe stores	Meal compositions. Study 2. Patties contained either: - 100 g beef; addition of 2.0 mg Fe (as FeCl_3_) prior cooking; or - 100 g beef + 30 g TSP	E(R) Beef meal: 5.9 mg, 2 mg as FeCl_3_, 3 mg native nonheme Fe + 1.2 mg heme iron Beef + soy meal: 5.9 mg, 1.2 mg as heme 1 μCi ^59^FeCl_3_ for nonheme Fe absorption 3 μCi ^55^Fe hemoglobin for heme Fe absorption determination	nr	Nonheme Fe abs: - Beef patty: 5.94 ±1 SE: 4.03; 8.74 - Beef + soy patty meal: 3.24 ±1 SE: 2.27; 4.62 Heme Fe abs: - Beef patty: 18.04 ±1 SE: 15.45; 21.06 - Beef + soy patty: 28.63 ±1 SE: 24.05; 34.09	Fe absorption ratios (Beef + soy/Beef meals) differed significantly (*P <*0.01).

Abbreviations*:* E, extrinsic labeling, I, intrinsic labeling (generally by hydroponic culture) (R), radioisotope; (S), stable isotope; AA, ascorbic acid: AA:Fe, ascorbic acid to iron molar ratio: F, female(s); FeCl_3_, ferric chloride; FeSO_4_, ferrous sulfate; FSF, full-fat soy flour; geo mean, geometric mean; I, intrinsic iron isotope label; ID, iron deficient; ISP, isolated soy protein; M, male(s); min, minute(s); nr, not reported; n.s., no statistically significant difference; PA, phytic acid; PA:Fe, phytic acid to Fe molar ratio extrinsic; SF, serum ferritin; SPC, soy protein concentrate; TSF, textured soy flour; WSB, whole soybean.

1 Participants were not deficient unless indicated otherwise.

2 Iron absorption is reported as geometric mean (%) (range), unless indicated otherwise.

**FIGURE 1:**
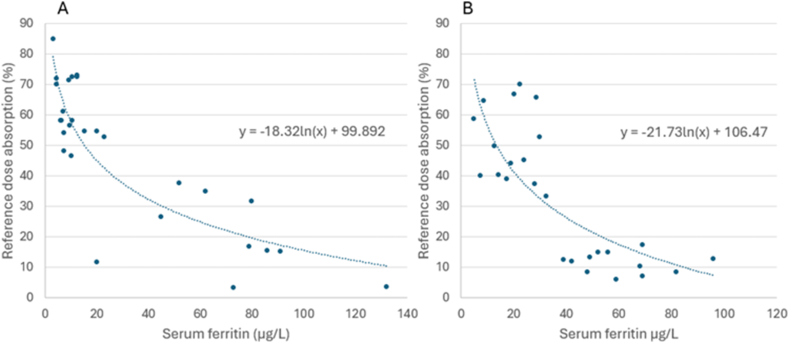
Relationship between reference dose absorption (RefA) and serum ferritin (SF) used to adjust iron absorption values from soy-based meals and semisynthetic meals in studies where serum ferritin was not determined, and a reference dose of ferrous ascorbate was provided. (A) RefA consisting of 3 mg Fe as ferrous sulfate (FeSO_4_) in water with 18 mg ascorbic acid (AA) (RefA = −18.32 ln SF + 99.89). (B) RefA consisting of 3 mg Fe as FeSO_4_ in water with 30 mg AA (RefA = −21.73 ln SF + 106.5).

**FIGURE 2:**
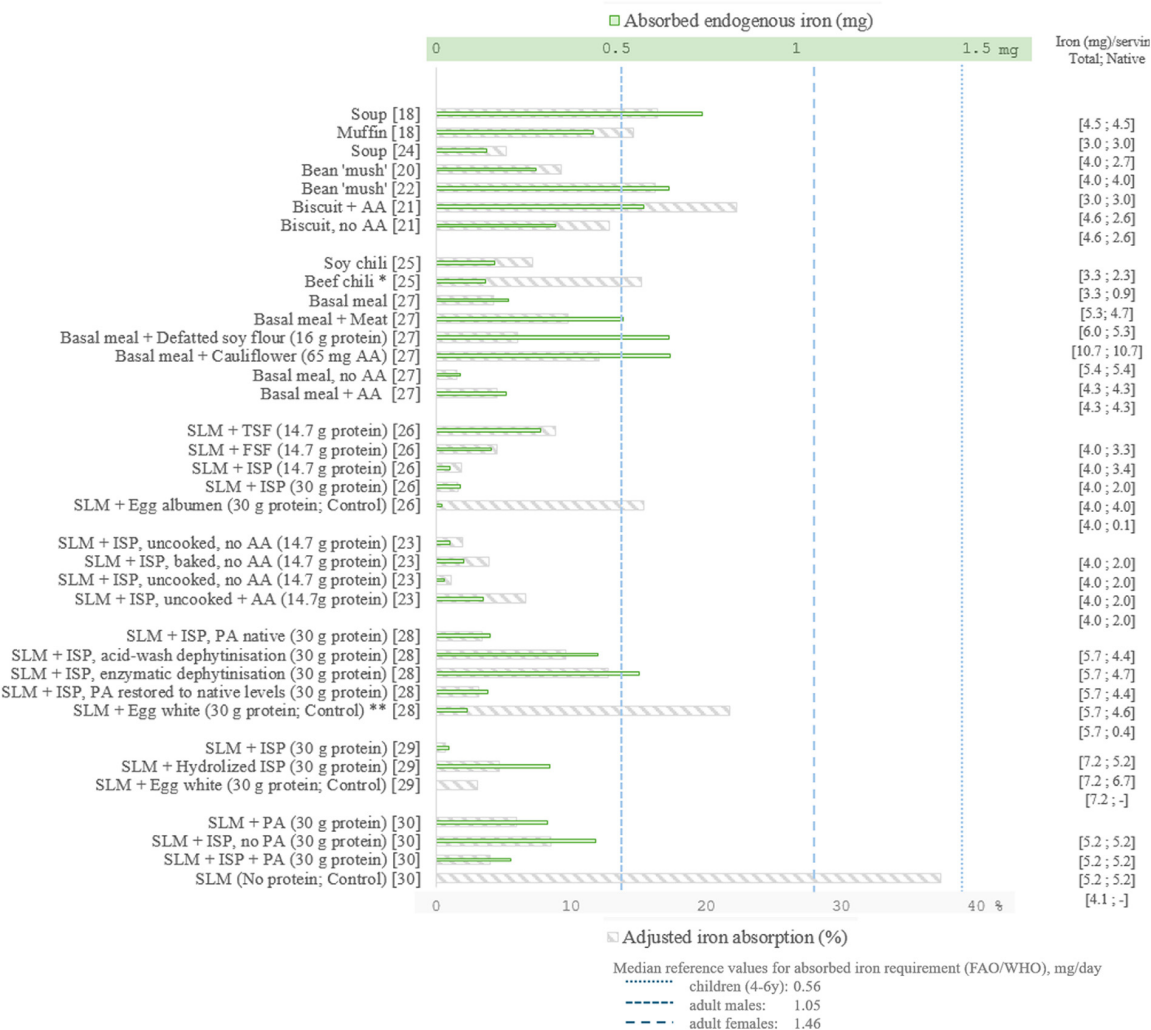
Adjusted iron absorption (%) and corresponding quantities of native iron (mg) absorbed from soybean-based meals and from SLM containing either TSF, FSF, or ISP. Gray bars depict adjusted iron absorption (%; geometric mean adjusted to serum ferritin of 15 μg/L), green bars depict absorbed native iron (mg) per tested meal; values on the right indicate total iron in the tested meal followed by the native iron contents from the soy ingredient (mg) in the corresponding meal; blue vertical dashes indicate median reference values for daily requirements for absorbed iron in children aged 4–6 y (that is, 0.50 mg Fe/d), adult males (that is, 1.05 mg Fe/d) and females (that is, 1.46 mg Fe/d) [11]. AA, ascorbic acid; N, nitrogen; PA, phytic acid; SLM, semisynthetic liquid meal, TSF, texturized soy flour; FSF, full-fat soy flour; ISP, soy isolated protein. ∗Data presented for beef chili [22] do not include absorbed heme iron. ∗∗Only average values are reported for data reported in [28].

As iron status is the main determinant of iron absorption, we standardized data pertaining to human iron absorption for iron status. The relation can be described mathematically by an inverse correlation with the use of ferritin as an indicator of iron status [8]. To improve comparability between studies, in those studies providing serum or plasma ferritin (SF) values, we standardized mean iron absorption values to a ferritin concentration of 15 μg/L (that is, a concentration mimicking the absence of iron stores) according to Cook et al. [38] and Armah et al. [10]. Several of the included studies did not use SF to characterize subject’s iron status but instead employed radioactive iron tracers using a reference dose of iron salts, that is, ferrous sulfate (FeSO_4_) combined with ascorbate served in water [17]. To standardize absorption values for iron status in those studies, and to maintain comparability, we fitted regression curves between SF and the reference dose absorption in those studies that assessed both SF as well as iron absorption from the ferrous ascorbate reference dose (Figure 1). We then adjusted human iron absorption values from the soy products to an SF concentration of 15 μg/L, via regression curves between ferrous ascorbate absorption and SF concentration to obtain an SF value. This value was then used to correct the reported absorption to an SF concentration of 15 μg/L [10] (Figure 2). The quantities of native iron absorbed from soy were also calculated to compare with daily requirements for absorbed iron in children, adult females, and adult males [11]. Only studies with nutritionally relevant quantities of native iron, that is, ≥2 mg iron per serving, are presented in Figure 2.

## Iron Forms Naturally Present in Soybeans

Iron moieties naturally occurring in plant foods are usually present in the ferric (iron III) oxidation state and are mainly present as ferritin (that is, phytoferritin), associated to phytate, or to a lesser extent to low molecular weight compounds [39,40].

Ferritin is an iron-storage protein that stores iron in a mineralized form inside its protein shell. It is present in virtually all living organisms, including bacteria, plants (that is, phytoferritin), and animals [41]. The most prevalent naturally occurring phytoferritins are found in soybean, pea, black bean, maize, alfalfa, and Arabidopsis [42]. Data on phytoferritin concentration in legumes is sparse and variable and may depend on genetic variation among plant varieties and/or environmental conditions [[43], [44]]. Furthermore, methodologies used to quantify phytoferritin require advanced analytical methods. Depending on the cultivar, several-fold differences in ferritin content have been reported in soybeans [45]. For example, ferritin iron quantification via exclusion chromatography and atomic absorption spectrophotometry in soybeans (variety not specified) indicated that 18% of the iron in soybeans was bound to phytoferritin [46]. However, isotope dilution mass spectrometry (conducted by the same research team) or Mössbauer spectrometry analyses showed that 38% or even 90% of the iron in soybeans was bound to phytoferritin, respectively [47,48]. Murray-Kolb et al. [18] produced hydroponically labeled soybean cultivars with ^55^Fe and determined precipitable and soluble fractions after tissue digestion. The protein (that is, phytoferritin) fraction contained 49.3% of the iron whereas the remaining iron was associated with soluble lower molecular weight iron forms. For comparison with other pulses, data generated by Hoppler et al. [46,48] are provided in Supplemental Table 1.

Iron solubility is considered a prerequisite for iron absorption, that is, the more soluble the iron, the higher its absorption would be. Iron from isolated, recombinant bean phytoferritin, and phytoferritin isolated from red kidney beans was released and was fully soluble under acidic gastric conditions [46]. Hoppler and Beard [45,46] reported that ∼39% and 52% of the total iron in soybeans was soluble in the evaluated species.

Soybean phytoferritin has been suggested as a bioavailable dietary iron source [49]. Lönnerdal et al. [50] isolated soybean phytoferritin to study iron absorption in healthy females without anemia. Participants received a standardized meal (that is, bagel, cream cheese, and apple juice) fortified either with radioactive FeSO_4_, the reference compound, or with iron-free soybean phytoferritin that was remineralized with labeled FeSO_4_ and reconstituted under a high phosphate condition (that is, iron:phosphorus of 4:1). On the basis of X-ray and Mössbauer spectroscopy, this reconstituted ferritin was indistinguishable from that in plant tissue. Iron absorption from the reconstituted phytoferritin was comparable with that from the FeSO_4_ reference (30% vs. 34%, no statistically significant difference) when consumed with the noninhibitory standardized meal. This suggests that phytoferritin from soybean is well absorbed when administered in a noninhibitory meal and that the difference in phosphate content of the iron mineral did not affect iron absorption. In addition, consistent with its predicted solubility (Supplemental Table 1), a significant inverse relation between SF level and the subjects iron absorption from the phytoferritin isolated from soy was found [50]. On the basis of these elements, the authors proposed phytoferritin fortification and/or ferritin expression as selection criteria for plant breeding, and as a promising approach to enhance iron content in staple crops [50].

Phytoferritin and other iron forms present in plant-based foods are classified as nonheme iron [40], indicating that these forms are impacted by iron absorption enhancers and inhibitors present in the diet [51]. However, past findings from a simulated digestion model coupled with caco-2 cells suggest that depending on the gastric pH the protein shell of the isolated phytoferritin may protect iron from interacting with inhibitors [49,51], even if there is currently no support from human studies for this suggestion.

Part of the iron that is present in soybeans is bound to phytate. Phytic acid, also called myo-inositol hexaphosphate (IP6), contains 6 phosphate groups that are attached to a myo-inositol ring. Among other physiological functions, phytic acid serves as a storage for phosphorous and other cations in the plant. It is generally accepted that phytic acid chelates metal ions at low pH and forms insoluble complexes with low bioavailability. Processing of unrefined cereals and legumes can reduce their phytate content through dephosphorylation of IP6 to lower myo-inositol phosphate forms [52,53]. Some of these dephosphorylated forms (that is, inositol mono- and diphosphate) are reported to no longer inhibit iron absorption [[52], [53], [54]]. Monoferric phytate as a fortificant has been shown to be water soluble and bioavailable in humans, rats, and dogs [[55], [56], [57]]. The inhibiting effect of other phytates (inositol tetraphosphate to IP6) on iron is dose-dependent and is present at low concentrations (Table 1). The addition of AA can counteract the inhibitory effect of phytic acid and other inhibitors of iron absorption [8], increasing the absorption of both ferrous (Fe^2+^) and ferric iron (Fe^3+^) in a dose-dependent manner [8,[58], [59], [60]]. To optimize iron absorption from foods, WHO/FAO guidelines recommend molar ratios of phytic acid to iron (PA:Fe) as well as AA to iron (AA:Fe) [61]. For PA:Fe, the guidelines suggest <1:1, or even <0.5:1, to achieve a meaningful increase in iron absorption in the absence of iron absorption enhancers [61]. To enhance iron absorption, the addition of AA in an AA:Fe ratio of 2:1 or 4:1 (for foods high in phytic acid) is recommended [61]. Hurrell and Egli [8] proposed PA:Fe <1:1 in plain cereal or legume-based meals that contain no enhancers of iron absorption, and PA:Fe <6:1 in composite meals with certain vegetables that contain enhancers such as AA or meat.

## Iron Absorption from Soybeans Served as a Meal

We included 7 studies documenting the absorption of iron naturally present in soybeans consumed as a meal (Table 1). The studies were conducted between 1969 and 2011 and mainly employed intrinsic labeling of the plant (hydroponic culture) with radioisotopes. Four of these studies compared the intrinsic (I) and extrinsic (E) iron (I:E) labels to determine the iron absorption of the native iron contained in soy. These studies showed the equivalence of the 2 types of labels with I:E absorption ratios close to 1 [[19], [20], [21]]. Although extrinsic labeling may not be equivalent to the intrinsic approach in all types of cereals or legumes [20], ratios for soy are comparable and we, therefore, considered data generated with either labeling approach. The meals provided in the reviewed studies included soybeans prepared as bean mush, that is, puree [19,20,22,23], soup [18,24], muffin [18], or biscuit [20,21] (Table 1).

Iron absorption from soybean-based meals showed high overall variability, ranging between 1.7% and 27%, and was the highest among subjects with iron deficiency. We adjusted native iron absorption from soybeans served as a meal to 15 μg/L of SF in all studies, but 1 [19]. Native iron absorption after adjustment ranged from 4.1% to 22.2% (Figure 2). This wide range may have several reasons: *1*) differences in the food matrix, including both the soy cultivar as well as the cooking and/or preparation methods. *2*) Iron status adjustment did not account for different methodologies that were used to assess iron status across the different studies. *3*) Interstudy differences in administering the isotopic labels may have contributed to the heterogeneity of the results.

Only Murray-Kolb et al. [18] reported the concentration of phytic acid in the beans, and none of the reviewed studies reported on other potential iron absorption inhibitors (for example, polyphenols and calcium) in the studied soy-based meals. The PA:Fe for the soup (18.3:1) and muffin (13.7:1) were well above the recommended ratio for optimal iron absorption (1:1) in the absence of iron absorption enhancers [8,61]. The authors hypothesize that the high absorption could have been due to iron linked to phytoferritin (that is, 49% in this study) with the remainder being acid-soluble monoferric phytate and other soluble low molecular weight forms of iron.

Only Sayers et al. [21] assessed the impact of AA addition on native iron absorption from soy biscuits (AA:Fe after baking of 2.6:1) and reported no impact. However, the evaluation was carried out in 2 groups of subjects that differed in iron status, and when absorption was adjusted to a SF of 15 ug/L, it showed a 2-fold increase in native iron absorption in the presence of AA (Figure 2) [21].

Interestingly, the studies reporting the lowest iron absorption from native iron in soybeans [23,24] were performed in non-iron-deficient males, and the tested meals were prepared with several sources of polyphenols (quantities not reported) that is, diced onion, bay leaves, garlic powder, and red pepper. It is possible that methodological questions or uncharacterized factors in the test meals may explain the discrepancy with the results by Murray-Kolb et al. [18].

In studies employing nutritionally relevant quantities of iron per serving (that is, >2 mg native iron/serving), the corresponding levels of absorbed iron were sufficient to cover between 13% to 70%, and 10% to 40% of the daily requirement for absorbed iron in adult males and females [11], respectively (Figure 2).

The reviewed evidence shows that soybean can potentially be a relevant contributor to bioavailable dietary iron and that this can be improved in the presence of small amounts of AA. Future research should address the causes for the large variability between studies such as the effect of the soybean variants and meal composition. In addition, efforts should be made to better understand the impact of food ingredients containing polyphenols such as seasonings (for example, onion, garlic) and flavors (for example, vanilla extract) on iron absorption.

## Nonheme Iron Absorption from Native Iron in Soybean Derivatives Obtained by Processing

We evaluated the iron bioavailability of foods containing soybean derivatives such as defatted flour, soy concentrates, or isolates which were served: *1*) as part of a plant-based meal and compared with a meat-containing equivalent, *2*) as part of a semisynthetic liquid meal (SLM); or *3*) as meat extender.

We identified 2 studies comparing iron absorption from meals that contained either soy or meat as a main protein source. The first study assessed iron absorption from a chili (served with tomato, corn chips, and grated cheddar cheese) containing either 40 g ground beef (PA:Fe 3.5:1) or 52 g low-phytate soy concentrate crumbles (PA:Fe 3.7:1) in children aged 4–8 y that were iron replete (SF: geometric mean 30 μg/L) [25]. The total protein content per meal was 11.4 g. The total iron content was adjusted to 3.3 mg per serving, that is, beef chili meal contained 0.9 mg native nonheme iron and 2.4 mg added FeSO_4_; whereas the soy chili contained 2.3 mg native nonheme iron and 1 mg labeled FeSO_4_. No AA was added. Nonheme iron absorption was significantly higher from the beef (7.6%) compared with the soy (3.6%) chili [25]. The adjusted iron absorption to an SF of 15 μg/L corresponded to 15.2% and 7.1% for the native nonheme iron present in the beef and soy, respectively. Considering the similar PA:Fe in the 2 meals, the authors attributed the lower absorption from the soy chili to inhibitors present in the soy concentrate. The percentage of absorption from this study falls in the lower range of values retrieved for soybean when consumed with seasonings (aforementioned section) [24,26]. In the study, each chili meal contained 8 g/person of mild chili, which may have contributed to the low iron absorption in the soy chili meal, in the absence of the absorption-enhancing effect of meat [8]. Previously, chili powder added to iron-fortified rice was reported to reduce iron absorption by 38% (6.0% vs. 9.7% without chili) [62]. Although meat improved iron absorption, the quantity of native nonheme iron in soy was higher than in meat, and the corresponding net quantities of nonheme iron absorbed from soy and from meat were similar (Figure 2). A limitation of this study is that the heme-iron moiety was not labeled and its absorption was not determined, impeding our ability to compare the total quantity of iron absorbed in both meals. In addition, in the meat-based chili, nonheme iron in the form of FeSO_4_ was added introducing a further limitation to this comparison.

The second study consisted of 4 tests investigating the impact of various meal components that were added to a Latin-American-type meal (basal meal) consisting of maize chapattis, black beans, and rice in subjects without anemia [27]. The components that were added to the basal meal included 75 g of meat (ground beef; study 1), 33 g defatted soy flour (study 2), 65 mg AA from 125 g of cauliflower (study 3), or 50 mg added AA (study 4). The protein content (15 g per serving) of the meat and soy flour was equivalent. The basal meals contained 4.7 mg nonheme iron, the meal with added meat contained 5.3 mg iron (including 0.7 mg heme iron). Basal meal with defatted soy flour, cauliflower (AA:Fe of 1.9:1), and added AA (AA:Fe of 3.7:1) contained 10.7 mg, 5.4 mg, and 4.3 mg nonheme iron, respectively. Unadjusted iron absorption from the basal meals was 3.2%, 6.0%, 4.0%, and 1.2% in studies 1–4, respectively. Iron absorption from meals containing meat, soy flour, cauliflower, and added AA was 8.4%, 4.8%, 10.8%, and 2.8%, respectively (adjusted value: 9.8%, 6.0%, 12.1%, and 4.5%). The addition of AA as such or through cauliflower increased iron absorption by 2–3-fold to the same extent as the addition of meat, whereas the addition of soy flour marginally decreased iron absorption. Absorbed quantities of iron from the basal meal with meat and with soy were similar, likely owing to the effect of meat on nonheme iron absorption and to the high iron content of defatted soy flour (Figure 2). Of note, in study 4, different raw material batches were used for the basal meal. These may have contained higher levels of iron absorption inhibitors which may explain the relatively lower iron absorption levels compared with the other meals that were assessed.

## Iron Absorption from Semisynthetic Meals Containing Soy Flours, Concentrate, or Isolate

Five studies investigated iron absorption from soy-based SLM (Table 1). In a series of studies, Cook et al. [26] investigated iron absorption in iron-replete male from uncooked SLM that were based on dextrimaltose-derived carbohydrates, fat from corn oil, and 2 levels of protein from different sources. The protein sources were full-fat soy flour (FSF), textured protein obtained from extruded defatted soy flour (TSF), isolated soy protein (ISP), and an iron-fortified egg white albumin control. In the first study, iron absorption from a meal containing 30 g protein from ISP had a significantly lower iron absorption compared with the ferric chloride (FeCl_3_) fortified egg reference (unadjusted: 0.46% vs. 2.49%; adjusted for SF: 1.65% vs. 8.80%). The second study compared iron absorption from 4 different beverages (containing FSF, TSF, ISP, or albumin) with half the protein content (14.7 g) compared with the first study. Interestingly, halving the level of ISP in the SLM had no impact on iron absorption (adjusted value: 1.91%). In addition, iron absorption was highest in TSF meals followed by FSF meals (adjusted values 8.8% and 4.51%, respectively).

Similar results were reported by Morck et al. [23], where ISP was added to an uncooked SLM (containing 14.7 g of protein) made from corn oil and carbohydrate. The authors showed that baking the ISP-fortified drink increased native iron absorption by 2-fold and the addition of AA (100 mg) by ∼5-fold. The finding of an iron absorption increase upon cooking is surprising as phytic acid degradation during cooking is generally considered marginal, and it is possible that this difference can be ascribed to other physiochemical changes during processing. However, depending on the type of food matrix, decreases of phytic acid have broadly been described to range between 10% and 30% in closed systems such as extrusion or autoclaving, and may be affected by the cation and protein environment, which in turn can be affected by heat treatment or other processing operations [63].

To optimize iron absorption from soy-derived ingredients, Hurrell et al. [28], evaluated the impact of dephytinization on iron absorption from several ISPs served as SLM that were mainly based on corn carbohydrates and fat with vanilla extract. Dephytinization was either performed by continuous acid-salt-washing and ultracentrifugation or by enzymatic treatment. The ISP-based SLM was tested in individuals across a wide range of SF concentrations (11–138 μg/L) with a fortified egg white drink serving as control. The study showed that dephytinization to PA:Fe <1:1 increased iron absorption ≤4.8-fold. Dephytinization followed by subsequent restauration of phytic acid to native levels returned iron absorption to the same level as without dephytinization. Except for 1 case, iron absorption from the soy SLM after dephytinization was still significantly lower than from the iron-fortified egg albumin control drink. The authors conclude that decreasing phytic acid in a meal from 220 to 110 mg (that is, PA: Fe 3.7:1 and 1.86:1, respectively) would have little effect on iron absorption, whereas a decrease to <10 mg/meal (PA:Fe <0.17:1) would improve iron absorption substantially. The authors further suggest that phytate-free soy protein isolates inhibit iron absorption due to iron that binds to insoluble soy peptides. This aspect has been further evaluated by Lynch et al. [29] who investigated iron absorption in iron-replete subjects from a similar SLM containing 30 g of protein as either soybean isolate or an egg white protein (control). The study found low iron absorption (0.28%) from unmodified soybean protein isolate (1.70% phytic acid), which starkly increased after an extensive enzyme hydrolysis of the soy protein source with low-phytate content (0.23 and <0.05% phytic acid). The increase was linked to the extent of the protein hydrolysis, with an absorption increase of 1.86% and 5.33% for an amino nitrogen to total nitrogen ratio of 0.19 and 0.47, respectively. For the 0.47 ratio, iron absorption significantly differed and was higher compared with the control (that is, 5.33% vs. 0.28% without hydrolysis). Additional experiments with purified proteins indicate that the decreased absorption could not be attributed to the glycinin, but rather to the conglycinin-related moiety independent of the phytate content [29]. Glycinin and β-conglycinin are the 2 major components of soy protein and constitute 65%–80% of the protein fraction of soy [29].

Similarly, Reddy et al. [30] assessed iron absorption in iron-replete subjects from the same type of SLM but with different protein sources, that is, 30 g of protein provided as iron-fortified egg white, meat, or dephytinized ISP. Iron absorption was only about a half or a quarter from the egg white (9.67% vs. 21.7%; SF: 34 μg/L) and dephytinized soy isolate protein SLM (2.6% vs. 11.44%; SF: 49 μg/L), respectively, when compared with the beverage without added protein. Meat addition increased iron absorption by 2.5-fold (to 26.7%), whereas the addition of 300 mg phytic acid to the SLM-containing ISP (PA:Fe: 4.9:1) drastically decreased iron absorption from SLM to about one-tenth (21.7% vs. 2.15%). The impact of added phytic acid was less pronounced in the dephytinized soy isolate compared with the other protein-fortified formulations (that is, with egg). The authors hypothesize that this reflects a lower baseline absorption of the ISP SLM compared with the other meal types and suggest that apart from phytic acid specific proteins present in soy would decrease iron absorption. It is important to note that most of the studies reviewed in this section were performed with 30 g of protein from ISP (added to 115 mL of the synthetic beverage), translating to ∼41 g protein in 200 mL serving of soy-based beverage. Although this may be relevant to specific products like infant formula (not reviewed here), the typical levels of soy protein per serving of a commercial soy drink or soy burger for the general population range from ∼8 to 12 g [64]. In addition, most of these products are made of soy flour or concentrate with a different protein concentration than soy isolate, and depending on the production process, soy isolate may have a ratio of conglycinin to glycinin that is higher than in flours and concentrates [65].

The impact of iron absorption enhancers (that is, AA) on native iron absorption from soy-based raw materials was demonstrated by Sayers, Hallberg, and Morck [21,23,27]. Macfarlane et al. [66] also demonstrated that adding 20 mg AA to an iron-fortified ISP-based (fully hydrolyzed) SLM (AA:Fe 1.6:1) more than tripled the absorption of native and fortification iron. Istfan et al. [31] measured iron absorption using isotope fecal mass balance in 6 adult males receiving a fully standardized diet with soy protein concentrate as the sole dietary protein source (0.8 g/kg/d) over the course of 82 d. Each meal contained 0.3% phytate (PA:Fe of 1.8:1) and was supplemented with 75 mg AA (AA:Fe 5.6:1). On average, subjects consumed 11.8 mg iron per day coming solely from the soy formula with a mean iron absorption of 13% (range: 9%–21%). This translates to 1.5 mg absorbed iron per day, which should cover the median daily requirement for absorbed iron in healthy males (1.05 mg). However, the authors report a decline in SF over the 12-wk study period of ∼20 ng/mL, which they attribute to the blood withdrawals (480 mL/subject over the study period) rather than to the consumption of the soybean formula.

After adjustment for iron status (Figure 2), the corresponding quantities of absorbed iron are found to contribute to the daily absorbed iron requirement by 14.6% and 10.5% with the SLM-based on FSF [26], 26.1% to 61.6% and 10% to 44.3%, with the SLM-based on defatted soy flour [27,66], and 27.7% and 19.9% with texturized defatted soy flour in adult males and females, respectively [26]. The observed wide range for defatted soy flour may be explained by the 2-fold difference in the iron content per serving reported in the 2 studies. In the study evaluating texturized soy, the iron content was lower than in the other ingredients, which suggests that texturization may support iron absorption. The chili based on soy concentrate contributed to ∼30% of the children’s requirements for absorbed iron [25]. These contributions may even be higher if such an ingredient was coingested with foods containing AA [31].

For SLM-based on uncooked ISP, contributions to the requirement for absorbed iron were lower but increased on dephytinization, cooking, or addition of AA [23,26,28,30] (Figure 2).

This limited evidence suggests that soy flours (including in texturized form), and concentrates could be considerable contributors to the absorbed iron requirements, which can be improved after dephytinization and/or co-ingestion with AA. The contribution of ISP to the dietary iron requirements appears to be considerably lower compared with that of soy flour or concentrates, which may be linked to its protein level and profile, and potentially to lower native iron concentration. Further investigation of these ingredients is needed to understand the impact of processing used for producing soy protein concentrates and isolates as well as their impact on the final product. Furthermore, the physical characteristics and the digestibility of texturized protein may play a role in iron bioavailability and merit further investigation.

## Nonheme Iron Absorption from Meat Extended with Soy Proteins

Five studies assessed nonheme iron absorption from meat-based meals in which meat was partially replaced by soy protein. The substitution levels for protein ranged from 20% to 50%.

Morris et al. [32] assessed iron absorption in non-iron-deficient males (*N* = 49) from a burger meal with patties that either contained beef only or in which 20% of beef was replaced by soy isolate, soy concentrate, or soy flour. Iron absorption was determined at baseline and after once to twice daily consumption of the soy-meat patties over a period of 180 d. Unadjusted mean nonheme iron absorption from the different meals was generally low and ranged from 0.9% to 1.04% at baseline from patties containing soy isolate and beef. In individuals with low iron stores, iron absorption from the soy-based meal increased to ≤7%. On adjustment by the study authors for the level of iron stores to 40% of the reference dose, the mean relative absorption of nonheme iron from meals containing soy isolate (7.5%) or soy flour (5.8%) even exceeded absorption from the beef-only patties (5.3%). The study has methodological limitations in that the iron tracer was delivered via an accompanying milk drink and not integrated into the patty, which may have impeded the full mixing of the radiolabel with the meal. Nevertheless, the impact on iron stores as indicated by the absorption dose of ferrous ascorbate and the measurement of SF after 180 d of the soy extended meat meal consumption showed no detrimental effects on the iron status of iron-replete males [32].

Sandström et al. [33] compared apparent iron absorption in 8 ileostomy patients who were randomly assigned to receive diets containing either meat, rice, and bread protein, or a 25% replacement of the protein with soy flour, soy concentrate, or soy isolate. The diets provided 60 g of protein per day and the 25% replacement by soy protein was achieved by replacing 50% of the protein in bread and 30% of the protein in meat. Iron intake increased in those subjects receiving the soy diets, whereas apparent iron absorption did not differ significantly between the diets [33].

Woodhead et al. [34] investigated iron absorption in school-aged children who were non-iron deficient and consumed lunch meals either containing a beef or a beef-and-soy patty (70% beef-30% soy) on 3 consecutive d. Mean iron absorption differed significantly between the meals and was higher in the beef compared with the beef-soy patty (2% vs. 1%); The authors underlined that such difference would not be nutritionally relevant.

Hallberg et al. [35]assessed the impact of soy flour that substituted 50% of meat in a hamburger meal with and without dephytinization. They found that reducing the meat content by 50% (that is, 7 g of meat protein with 7 g of texturized soy protein) led to a decrease in fractional nonheme iron absorption from 12.9% to 8.2%. The total amount of absorbed nonheme iron was unchanged, owing to the high iron content in the soy flour. Dephytinizing the defatted soy flour used to substitute 50% of the meat had no impact on iron absorption, potentially due to the low initial PA:Fe (1.5:1) [35].

Cook et al. [26] assessed iron absorption in healthy iron-replete male subjects (*n* = 36) from 3 different meals containing a patty with a bun, French fries, and a milkshake. The patty contained either 100 or 70 g lean ground beef with 30 g added TSF (moistened with water before mixing with beef; meat-to-soy-protein-ratios: 1.2 and 0.8, respectively) or 100 g beef only. Mean iron absorption from the meal containing no soy (3.2%) decreased significantly on the addition of TSF (1.24%; 1.51%) and was 61% and 53% lower in meals with the 1.2 and 0.8 meat-to-soy-protein ratios, respectively, compared with the meal without TSF.

Lynch et al. [36] partially replaced meat in a meat patty with soy protein to investigate nonheme and heme-iron absorption in iron-replete healthy male volunteers. The administered meals contained 100 g lean broiled beef, a white bun, French fries, and a vanilla milkshake (4.2 mg iron/meal, 1.2 mg as heme) or the same meal in which the patty consisted of 70 g beef and 30 g soy flour (5.0 mg iron/meal, 0.8 mg as heme). Unsurprisingly, the addition of soy flour to the meal decreased nonheme iron absorption substantially, however, it simultaneously increased heme-iron absorption from 33.1% to 42.1%, which the authors could not explain. This may be due to an exchange between the 2 labels (used to tag heme and nonheme iron) in conjunction with the meat factor—or may have other reasons. When analyzing the total amount of iron absorbed from the different meals, the authors concluded that adding soy flour (1/3) to a beef meal caused a modest decrease in iron absorption, due to both an increase in total iron in the meal as well as an increase in heme (label) iron absorption which partly offset the lower heme-iron content in the meals [36].

This scarce evidence aligns with an International Nutritional Anemia Consultative Group (INACG) report (1982) that states that substituting ≤30% of meat with soy protein in industrialized countries should pose few problems relative to iron nutriture. Nevertheless, this may also depend on the iron status of the population and on daily dietary iron intake levels. The INACG also concludes that absorbed iron would decrease proportionally to the degree of meat substitution and that this could partly be attributed to a decrease in a meal’s heme-iron content, a decrease in the enhancing effect of meat on iron absorption, as well as an inhibitory effect of soy [67]. This is also shown by studies in which >30% of meat within a meal is substituted with soy and where nonheme iron absorption tended to decrease.

## Discussion

This review synthesizes evidence on iron bioavailability from isotope studies conducted in human, predominantly adult, populations. The findings show that native iron from soybeans, soy flours (including texturized), and soy concentrates, and to a smaller extent isolates, can represent a relevant source of absorbable dietary iron. Dephytinization and/or coconsumption with AA drastically increased the absorption of native iron from all considered soy matrices. The iron-storage protein phytoferritin provides bioavailable iron and has been suggested as a promising means for biofortification and potentially fortification purposes.

Although we found no studies investigating the impact of iron absorption from soy-based food over time, we identified 3 longitudinal interventions that assessed the impact of soy-based food on iron status (details are provided in Supplemental Table 2). These studies provided ∼28%, 50%, and 50% of protein via soy to females in pre-, peri-, and postmenopause, respectively, and consistently showed no impact on iron status in the soy-consuming groups [[68], [69], [70]]. Soy protein in 2 studies [68,69] was provided as ISP and from multiple sources in the third one [70]. Interestingly, the third study only showed a decline in iron status in females in postmenopause when the soy product was consumed with its native PA:Fe level (10–12:1) but not at lower PA:Fe levels (3.5:1) [68]. This is in agreement with INACG 1982 [67], which indicates a limited impact on iron nutriture on replacing <30% of meat protein with soy, especially when consumed with AA and when PA:Fe is <6:1. Among the limitations of the review is that we did not perform a systematic literature search. Most of the reviewed evidence stems from studies in generally healthy, iron-replete subjects and was generated in the last century. Methodological differences between the studies for which we were unable to control exist. These include, for example, the employed labeling technique and potential residual confounding due to individual differences between participants unaccounted for by the iron status correction. A major strength of this review is that we adjusted for SF and reference dose absorption where possible. Although this allowed us to reduce variability among the studies, large heterogeneity among the results remained, which cannot solely be attributed to differences in participants’ iron status. It is also worth emphasizing that although correction to 40% of the reference ferrous ascorbate absorption was developed with AA:Fe (3.2:1) provided in 10 mL water [17], we observed several deviations from this protocol. Some more recent studies report high levels of iron absorption from soybean-flour-based meals, and these results contrasted with findings from earlier studies. It is possible that besides iron status and/or other parameters outlined previously, differences in the tested soy food matrices across studies could be 1 factor. This reflects the high versatility of soy-based products and their application in the food supply.

In conclusion, soy-based products are a heterogeneous class of products for plant-based and omnivorous diets and with the potential to provide a high-quality protein source that is also rich in iron. Iron absorption studies focusing on soy products suggest that they can provide a valuable natural contribution to the absorbed iron intake, especially if measures are taken to reduce phytic acid and when consumed with AA. The high heterogeneity of results calls for further studies investigating factors that affect iron bioavailability from iron naturally present in soy-based products.

## Author contributions

The authors’ responsibilities were as follows – MS: defined review topic, screened articles, and synthesized evidence; and all authors: wrote the paper, have primary responsibility for final content and read and approved the final manuscript.

## Funding

This research was funded by Nestlé Product Technology Center Lebensmittelforschung GmbH, Singen, Germany.

## Conflict of interest

MS and LH report a relationship with Nestlé Research & Development that includes employment. DM reports a relationship with Nestlé Research & Development that includes: consulting or advisory and declares no conflict of interest. His institution (SUPSI/FFHS) received an honorarium for his contribution consistent to the time invested and rates stipulated by SUPSI for his degree and work class. LH and MS are Nestlé employees.
