# Supplementary material for: Absorption of Iron Naturally Present in Soy
Source: Adv Nutr. 2025 Feb 26;16(4):100396. doi: 10.1016/j.advnut.2025.100396 (PMC12008532; doi:10.1016/j.advnut.2025.100396)
Supplement: multimedia component 1 [file mmc1.docx]

**Absorption of iron naturally present in soy**

Hackl et. al.

**Supplementary** **Table 1**: Mean iron concentration (µg/g) and iron distribution (% linked to ferritin) in selected uncooked legumes, modified from Hoppler et al. [46, 48]. Specifics on tested varieties were not provided.

| **Legume** | **Total iron concentration** | **Ferritin- bound** | **Other soluble iron** | **Total soluble iron** |
| --- | --- | --- | --- | --- |
|  | **µg/g, mean ±SD** | **% of total iron** | **%** | **%** |
| Soybeans [46] | 72.4 ±1.3 | 18 | 34 | 52 |
| Soybeans [48] | 65.8 ±0.9 | 38 | - | - |
| Dry pea [46] | 47.8 ±0.8 | 42 | 31 | 73 |
| Green pea [48] | 45.2 ±1.7 | 52 | - | - |
| Yellow pea [48] | 47.8 ±0.8 | 62 | - | - |
| White beans [46] | 50.2 ±0.7 | 25 | 65 | 90 |
| Red kidney beans [46] | 62.4 ±0.5 | 20 | 44 | 64 |
| Red kidney beans [48] | 64.4 ±1.0 | 15 | - | - |
| Pinto beans [48] | 55.6 ±0.6 | 29 | - | - |
| Lentils [48] | 59.4 ±1.2 | 69 | - | - |
| Recombinant bean ferritin [46] | 47.8 ±0.8 | 100 | 0 | 100 |

**Supplementary Table 2:** Studies reporting on the impact of long-term consumption of soy-based products on iron status.

| **Ref** | **N** | **Tested conditions** | **AA** | **Fe content (mg/d)** | **Impact on Fe status** |
| --- | --- | --- | --- | --- | --- |
| Zhou et al 2011 [70] | 63 (2 groups) | Iron status measured in pre-menopausal women (18-28y) following a semi controlled meat or soy-based diet for 10 weeks.   - In soy-based diets » 2 servings per day were substituted with soy-based products, e.g., yogurt based on soy protein isolate (ISP) vs conventional yogurt, soy chili vs meat chili, soy milk vs cow’s milk, soy burger vs meat burger, soy meat balls vs meat balls, edamame vs green peas, etc.   Average soy protein content in the soy group : 19 g/day.  No information on phytic acid content was provided.  Molar ratio ascorbic acid to Fe (AA:Fe) »2.3:1 (based on daily intake) | » 98 mg/d (in both groups at baseline | Meat diet: 13.7  Soy diet: 14.0 (baseline) | No significant difference between baseline and post intervention. |
| Swain et al 2002 [69] | 69 (3 groups) | Peri-menopausal women (50 y in average) were assigned to isoflavone rich soy protein isolate (ISP), isoflavone poor-ISP, or whey protein (control); 40g of protein/day consumed over 24 weeks; 50% of the protein powder was incorporated into a muffin, 50% incorporated as a powder by the subjects in food or beverages (powder was consumed as meal replacement and not as a supplement).  No information on phytic acid level was provided.  AA:Fe » 2.7:1 (based on daily intake) | »117 to130 mg/d | » 13.5 to 15.8 | No significant effect on iron status. |
| Hanson et al 2006 [68] | 52 (2 groups) | Healthy post-menopausal women (47-72y) consuming an ISP (40g/day) with low or native phytic acid (and w/wo isoflavone) for 6 weeks.  2 sachets a day (20g ISP/sachet) to be consumed with meals and/or beverages.  PA:Fe 3.5:1 (low) versus 10 to 12:1 (native phytic acid).  Results from the two groups testing the product w/wo isoflavone were not considered. | Not reported | 5,6 mg Fe brough by formula | Significant decrease of iron status in group consuming ISP with native phytic acid content. |

AA: ascorbic acid; AA:Fe: Molar ratio AA to iron; ISP: soy protein isolate.
